# A qualitative study investigating Australian cancer service outpatients’ experience of distress screening and management: what is the personal relevance, acceptability and improvement opportunities from patient perspectives?

**DOI:** 10.1007/s00520-021-06671-2

**Published:** 2021-11-25

**Authors:** Kristen McCarter, Melissa A. Carlson, Amanda L. Baker, Chris L. Paul, James Lynam, Lana N. Johnston, Elizabeth A. Fradgley

**Affiliations:** 1 School of Medicine and Public Health, University of Newcastle, Callaghan, Australia; 2 Calvary Mater Hospital Newcastle, Hunter New England Health, Waratah, Australia

**Keywords:** Cancer, Distress, Implementation, Quality outcomes, Psycho-oncology

## Abstract

**Purpose:**

People diagnosed with cancer experience high distress levels throughout diagnosis, treatment, and survivorship. Untreated distress is associated with poor outcomes, including worsened quality of life and higher mortality rates. Distress screening facilitates need-based access to supportive care which can optimize patient outcomes. This qualitative interview study explored outpatients’ perceptions of a distress screening process implemented in an Australian cancer center.

**Methods:**

Adult, English-speaking cancer outpatients were approached to participate in face-to-face or phone interviews after being screened by a clinic nurse using the distress thermometer (DT). The piloted semi-structured interview guide explored perceptions of the distress screening and management process, overall well-being, psychosocial support networks, and improvement opportunities for distress processes. Thematic analysis was used.

**Results:**

Four key themes were identified in the 19 interviews conducted. Distress screening was found to be generally acceptable to participants and could be conducted by a variety of health professionals at varied time points. However, some participants found “distress” to be an ambiguous term. Despite many participants experiencing clinical distress (i.e., DT ≥ 4), few actioned referrals; some noted a preference to manage and prevent distress through informal support and well-being activities. Participants’ diverse coping styles, such as positivity, acceptance, and distancing, also factored into the perceived value of screening and referrals.

**Conclusion and implications:**

Screening models only measuring severity of distress may not be sufficient to direct care referrals, as they do not consider patients’ varying coping strategies, external support networks, understanding of distress terminology, and motivations for accessing supportive care services.

**Supplementary Information:**

The online version contains supplementary material available at 10.1007/s00520-021-06671-2.

## Introduction

People with a cancer diagnosis experience distress at higher levels than that of the general population during diagnosis and treatment [1]. The National Comprehensive Cancer Network (NCCN) defines distress as “a multifactorial unpleasant experience of a psychological (i.e., cognitive, behavioral, emotional), social, spiritual, and/or physical nature that may interfere with the ability to cope effectively with cancer, its physical symptoms, and its treatment” [2]. Surveys have indicated that up to half of people with a cancer diagnosis experience significant levels of distress [3–6]. If left untreated, this distress can lead to poor outcomes including decreased social functioning, increased intensity in physical symptoms, cognitive impairment, poor adherence to treatment, and reduced length of life [7–10]. As such, distress has been branded and is recognized internationally as the sixth vital sign [11, 12]. As distress levels peak during diagnosis and initial treatment stages [1], it is important to recognize and implement effective distress screening management processes within treatment facilities where there is an opportunity for early intervention.

*Distress screening and management is beneficial, but implementation challenges remain:* Timely and standardized distress screening if coupled with well-structured psychosocial referral systems can reduce patients’ emotional distress and improve their quality of life [3, 13, 14]. The benefits also extend to reduced physical symptoms and improved satisfaction with care and communication between patients and professionals [2]. There is also evidence that psychosocial screening reduces risk of emergency service use and hospitalization [15].

There remain challenges to the routine use of distress screening especially in time-poor clinical services. Firstly, there remains debate on the utility of single-item distress screening tools, such as the distress thermometer (DT) [16], especially without the use or availability of a well-structured referral pathway [16, 17]. Secondly, the implementation of distress screening programs is poorly reported, and it is likely that only select components of evidence-based approaches are being incorporated in health services, such as one-step screening or no rescreening [18, 19]. These two factors may have contributed to emerging reports that health professionals and services are unclear on the potential benefits of distress screening programs and thus unable to rationalize both the real and opportunity cost of yet another clinical activity [20].

*Discrepancies between patient-reported acceptability and professional perceived acceptability exist:* Australian data suggest the majority of cancer service representatives felt patients did not want to be asked questions about their distress, with 38% of health services reporting that they never, or rarely, screen for distress [21]. However, a quantitative study of 498 patients’ experiences with a distress screening program implemented in ten Dutch hospitals found that patients’ evaluations of the process were largely positive [22]. Opinions were more favorable in patients who more frequently completed the DT and problem checklist and were exposed to information about the tools and a discussion of potential referral options. In an Australian context, a cross-sectional study surveying callers (*n* = 100) to a cancer helpline reported that over 74% of callers diagnosed with cancer were comfortable with DT use [23]. Given the potential discrepancy between patient-reported acceptability and professional-reported perceived acceptability, there is a compelling demand for in-depth exploration on how patients experience distress screening within Australian cancer services. Qualitative research provides an opportunity to provide context and further insight into existing and new distress screening processes [24].

This qualitative study explored the lived experiences of distress screening and management from patient perspectives through the use of semi-structured interviews with people with a cancer diagnosis. The research aim of this study was to qualitatively evaluate a rapid distress screening process in very early implementation phases and to collate patient perspectives on the acceptability and role of standardized screening in managing their overall emotional well-being.

The study was conducted in a large tertiary outpatient cancer service during the rollout of a cloud-based distress screening tool. The distress screening procedure was designed by the health service to be rapid, administered at each clinical appointment, and integrated into electronic medical records. This study recruited an opportunity sample of the patients screened and provides insight into how patients’ perceive the value and approach of brief screening models in Australian health services as part of their overall cancer journey.

## Methods

### Study design and recruitment

The qualitative interview study is reported in accordance with the consolidated criteria for reporting qualitative (COREQ) guidelines [25]. Patients were approached by a clinic nurse and invited to participate between May and July 2019. The screening pathway included asking all cancer outpatients to complete the DT and problem checklist on a kiosk before their appointment. The DT has an 11-point scale ranging from 0 (no distress) to 10 (extreme distress) [3]. The problem checklist prompts the patient to identify sources of distress using a problem list. Each item is directly related to one of five domains: practical, relationship, emotional, spiritual, or physical. Inclusion criteria included being at least 18 years of age, proficient in English, and having received a cancer diagnosis. In line with best practice guidelines that recommend all outpatients are screened for distress regardless of demographic or clinical characteristics, there were no exclusion criteria applied to time since diagnosis, treatment status, or cancer type for the interviews. The research team recruited participants using a purposive sampling approach to ensure representation of gender and a broad age range. The project was approved by Hunter New England Human Research Ethics Committee (2018/ETH00520).

### Data collection

A research team member (MC) with experience in qualitative research conducted 19 semi-structured interviews (30–45 min). Interviews were conducted face to face (*n* = 4) or via telephone (*n* = 15). The semi-structured interview guide included questions pertaining to perceptions of the distress screening process, emotional well-being, reasons for referral uptake/non-uptake, experiences of accessing new or existing professional and personal psychosocial support networks, and ways the distress screening and management process could be improved. Individuals were asked medical and demographic questions during the interview to contextualize findings. Individuals were also asked to complete the DT at time of interview as a way to prompt recall of their in-clinic experience, and in the case, they were asked other screening questions by other services. Participants’ DT score also provided context when discussing experiences, for example, gaps in referrals for moderately to severely distressed individuals or perceived value of screening for mildly distressed individuals. The interview guide was pilot tested with three members of a consumer advisory panel.

The research team considered data collection to have reached saturation when information became repetitive or no new information was being obtained; this was agreed upon through review of transcripts and field notes and discussion with the research team (MC, KM, EF) [26]. Multiple meetings to review transcripts, comprehensiveness of codebook, and emergence of new themes (if any) were held over a 2-month time period. All participants declined the opportunity to review their transcripts.

### Data analysis

Audio recordings were transcribed verbatim and coded by two team members (KM, MC) using NVivo 12 qualitative data analysis software. Using an inductive thematic analysis framework [27], a sample of transcripts was open-coded prior to collaboratively developing a codebook. The remaining transcripts were coded in batches using an iterative process of discussion and codebook refinement. Following coding, clear themes were identified and related back to data extracts to ensure coherence.

## Results/findings

Of the 39 patients invited to the study, 29 eligible individuals consented to be contacted by the research team. The 10 individuals who declined to be contacted cited a lack of time or the perception that they had little to contribute to the study. Of the remaining 29 participants who consented to contact, 19 consented to interviews prior to saturation being reached. The demographic characteristics of 19 participants are listed in Table 1.

**Table 1:** Sample characteristics

Characteristic	*n* = 19	%
*Gender*		
Male	13	68
Female	6	32
*Age range (mean)*	30–78 (68)	
*Months since diagnosis*		
Less than 6	3	16
6–24	6	32
25 or more	10	53
Distress score on distress thermometer*		
0–3	6	32
4–10	13	68
*Cancer diagnosis*		
Bowel	5	26
Prostate	5	26
Bladder	1	5
Skin cancer	1	5
Head and neck cancer	2	11
Testicular	1	5
Paraganglioma	1	5
Kidney	3	16

*On the day of interview

Four overarching themes were derived from the data. In talking about distress screening procedures, many participants expanded their discussion to include their attitudes toward distress, well-being, styles of coping, and understanding of the term “distress.”

### Attitudes toward formalized screening and logistics

Quotes are provided for each of the subthemes in Table 2.

**Table 2:** Quotations: attitudes toward distress screening and logistics

Acceptability
“Oh it’s not a problem” (Male, 65, colon cancer, DT = 10)
“It’s good” (Female, 63, leukemia and esophageal cancer, DT = 6)
“No trouble. It only takes a couple of minutes, and you’re there.” (Male, 70, prostate cancer, DT = 10)
“I think it’s a good idea. As I said, (my doctor) wouldn’t have known that I was feeling any stress unless (the research assistant) did that computer thing.” (Female, 53, paraganglioma cancer, DT = 7)
“It’s not that I’m not a big fan. It’s just for me, I don’t know that there’s much value in it. Because I’m not the same, I’m probably going to give the same answers for most of the time. Unless of course, as my diagnosis goes on and things get worse, then maybe I might become more distressed.” (Female, 31, colorectal cancer, DT = 0)
“I’d answer it honestly. Instead of trying to make a nervous joke, I can just say, I am an eight on the distress scale today, and I am not thinking well, and things like that.” (Male, 30, testicular cancer, DT = 7)
“I don’t think sitting down at a computer, putting down… They’ll just put down anything they want to put down, on a computer. Who’s going to read it? …it would be better if the oncologist asked the questions. I don’t want to write anything down on a computer. I would’ve liked that -the physical intervention, where you’re asking me these things, now, why couldn’t they—somebody else, or one of the nurses, or even the doctor…” (Male, 75, bladder cancer, DT = 5)
“I’m probably not… I don’t know whether a huge fan is the best way to put it. I understand it has to be done, but for my benefit, I was thinking I’m not really distressed.” (Female, 31, bowel cancer, DT = 0)
Who to conduct screening
“Well, because we see the nurses and speak to them often, I suppose that would be a good start, yes. Because the surgeons or the team of doctors, we see them after longer periods like after three months or six months or things like that. The nurse widely handles all the treatment which is the critical time as I get the chemo or the radiation, so you will see them nearly every second week or something like that anyway.” (Male, 65, colon cancer, DT = 0)
“Well, it depends if my distress is related to what the oncologist is treating, then I think that’s… but if my distress is related to something else, then I’m sure the GP would make some recommendations, or talk about that… So, it depends what I’m distressed about, I think, as to who might be the best.” (Male, 70, prostate cancer, DT = 0)
“Probably [my social worker]. She asks questions straight away, how are you, and I’ll tell her. If the doctor asks, I’ll just tell him, but they don’t ask. So, I don’t know. It probably is the social worker or the counsellor.” (Male, 75, bladder, cancer, DT = 5)
When to conduct screening
“I still say any time. It’s an ongoing sort of thing… Like me, I hide a lot. So, people, they don’t really tell you things.” (Female, 53, head and neck cancer, DT = 7)
“I think after about a week or two weeks it should be okay. Because at the beginning, you’re just taken onboard and you don’t really know what’s happening…you have to go to this appointment and go to that appointment. But after about a week or two I think it would be good if they start asking how you are feeling about it about more issues like that.” (Male, 65, colon cancer, DT = 0) “I think there should be some sort of screening, maybe when it’s first confirmed… It’s a big thing to hear…And when you’re actually told, it is this, this is what’s going to happen… it’s a lot to take in when they first tell you. So that’s probably when it would be a good point to start doing it.” (Male, 30 testicular cancer, DT = 7)

#### Acceptability

The majority of participants reported both the mode of in-clinic electronic delivery and the experience of distress screening as being acceptable and appropriate even if not directly relevant to themselves. The DT was described positively as short, and participants appeared happy to have this process included as part of routine care. The process was perceived to facilitate communication, with participants suggesting it would help them to be more honest about their distress than if they had been asked a more general question about well-being.

However, some participants did not appreciate completing the DT on a computer. One participant felt that responses would be inaccurate and would not be acted on if asked using a computerized kiosk, preferring human interaction.

#### Distress screening logistics

No single health professional was identified consistently as to who should be responsible for screening for distress; nurses, GPs, oncologists, social workers, and counselors were all suggested by participants. Participants had varying views on timing and recurrence of distress screening. Some felt that routine screening at repeated time points was appropriate. However, for some patients, distress screening was seen as not useful at initial diagnosis or beginning of treatment, when they were feeling overwhelmed with information.

### Managing distress and well-being

This theme encompassed discussions around participants’ awareness of their own distress and the desire for and access to services to assist. This conversation arose as part of discussing the sequential steps of the distress management pathway: screening, discussion, referral, and service use. Independent of their distress severity or health service use, participants also discussed self-initiated activities of daily living or leisure as a way to improve overall well-being. Quotes are provided for the subthemes in Table 3.

**Table 3:** Quotations: managing distress and well-being

Discussion, referral and service use within health settings
“‘I would suggest, that people are given the opportunity to speak to somebody, knowing that they can say whatever they like” (Female, 53, head and neck cancer, DT = 7)
“It’s almost like they (nurses) were handpicked for us, the way they look after us, welcome us, sit with us before we go in, and take us and make sure our bloods are done. Look, it’s only little things that they do, but for us it’s very important. And to be able to talk with us, to just ask us how are we going.” (Male, 64, prostate cancer, DT = 4)
“I know it’s available but I probably wouldn’t take it at this point in time … So, I don’t feel like at this point, I really need it. I feel like I’m more likely to talk to my family than a counsellor about things if I’m upset. I would talk through it, but if I’m upset, I tend to talk through things with family, I guess.” (Female, 75, bowel and liver cancer, DT = 4)
“You’re doing your job, asking the questions. I’m answering as much as I can. What happens after this, I don’t know. I’m out the door…. ……Here’s a list of services. Now, they did this. They give you a list of everything that’s available. But, nobody sat down and said, oh, what’s this? I can’t even spell that word. I can’t pronounce it. What’s that… Who does that? What’s that about? Who’s… Who does that? Nobody goes through the list with anybody; they just give you the leaflet. You sort out what you want yourself, and that’s it. If somebody had have said these services are available and we’d like you to pick one, two, or three, five, I don’t care, pick something that’s going to help you, and go through and let…” (Male, 75, bladder cancer, DT = 5)
Social support
“And, sometimes, some people don’t want to hear about your illnesses, but you need to have someone there that you can relate to, that can. Luckily, I’ve got a couple of friends that have had cancer, so we can relate, but you really need to have a group where people can just go to regularly, to speak to people. If you had something, say, once a week, just where… A room, there, where a group can just go and talk.” (Female, 53, head and neck cancer, DT = 7)
“Counselling services are pretty much problem-based, not… I guess the term I’d use is they’re not conversational. A lot of people value friends where they can just sit down over a cup of coffee and have a yarn about this and that, but if I regard that on a hierarchy, that’s about where things should start. It should start with… A discussion could be had just around this is impacting on my daily life or raising X, Y and Z, but you got to see a doctor. There’s got to be some sort of malady they can put a number against.”
“Oh, I couldn’t do it without her. Sometimes I get panic attacks. Like I get a tightness across my shoulders and then I get a tightness…. If it goes to my chest I wake [my wife] up and she massages my back and after a few minutes I just relax again.” (Male, 72, bowel cancer, DT = 5)
“Yes, it is, backup is really important, and the ladies at home, they’re just as important as my family to me because they see me more than the family does, yes, they work, you know, my family, so they’re there every day and they’ll come and knock on the door, are you alright, come over and have a cup of coffee, you know, it’s just, it makes all the difference in the world.” (Female, 78, esophageal cancer, DT = 6)
“Yes. My sons are always here. They’re doing things for me. If I’m going out somewhere, they’ll… If I need something done, they’ll always do it for me. Friends are the same. They ring up to find out and see how I’m going. It’s good to have that sort of support.” (Male, 63, bowel and liver cancer, DT = 7)
“I have good family that support me, and friends, but I found that it was… Having the treatment… It was really good. I had really good support. But then, as soon as you finished the treatment and everything seems to be fine, that they don’t have as much interest.” (Female, 63, leukemia and esophageal cancer, DT = 6) “Well, I’ve got a spiritual belief, and I think that helps… I pray each time; I suppose that’s reaching out. But if I was in trouble, then I could talk to a minister or that sort of thing. But I’m not in trouble, so, I’ve got no need to do that.” (Male, 70, prostate cancer, DT = 0) “I am a spiritual person. I do go to church, and I believe. I have a strong faith, so that has got me through immensely with this. Keeping me strong… But spiritually, yes, my church and my faith, and my church family are a huge part of my life.” (Female, 64, renal cancer, DT = 4)
Activities for well-being and reducing distress
“‘I do go for walks most every day. So, you go for a walk and it clears up your head and your mind as well. And you sort of come back refreshed and it really feels pretty good.” (Male, 65, colon cancer, DT = 0) “I’ve got a hobby for… of amateur radio, and I use that quite a lot, when I’m feeling distressed or low. Amateur radio. And I just go to the radio and chat to a few of my mates, and, yes, it lifts me up, and I just get on with it.” (Male, 75, bladder cancer, DT = 5)
“I do a fair bit of art and stuff. So, I tend to do that… I find that a form of meditation sometimes..” (Female, 31, bowel cancer, DT = 0)
“I’d try to keep busy. So, I’m not thinking about what the problem is… so, get out in the garden… or with the birds.” (Male, 70, prostate cancer, DT = 0)
“…it is very important, yes, to get out there and keep yourself busy. Otherwise, you just sit around, and you start to have all sorts of silly thoughts. You can get negative pretty easy.” (Male, 67, prostate cancer, DT = 8)
“I guess I’d have to say there’s nothing more precious that you can give to somebody other than your time at this stage, when time is of high value. I feel like something beautiful that I’d make for somebody and give it to them, that’s more than just a lump of wood, glued together and shaped, and varnished. I’m giving them some of my time.” (Male, 64, prostate cancer, DT = 4)
“I help out with the activities and after that we sit down and have a talk or before we sit down, we have a talk and… Because the residents were very good, because they used to go down and they like to see me and see how I was, and that helped too.” (Female, 75, bowel and liver cancer, DT = 4)

#### Discussion, referral, and service use within health settings

When prompted to speak about supports for management of their distress, many felt that a referral to formal support services such as a psychologist, social worker, or support group was not relevant to them. Although these participants stated that they did not believe that a referral would be useful to them, they nevertheless endorsed distress screening as a potentially important communication tool in their relationship with health professionals. At the same time, participants strongly emphasized the need for open communication with cancer nurses outside of a more formalized pathway, and explanations of the various forms of support were essential when discussing referrals.

A small number of participants reported recognizing that they needed support and would have welcomed a referral. One patient suggested that although they did not feel they needed formalized support at this stage of their diagnosis, it could be helpful if their health declined.

#### Other forms of support to manage distress levels

Participants spoke of the importance of various types of support, social, spiritual, and formal/clinical support when managing distress. For some participants, this was seen as just as important as talking to a professional about their feelings. The need for support was also evident in discussion about practical assistance such as getting to appointments, cooking meals, and house cleaning. One participant suggested that informal social support can wane after treatment has finished.

#### Activities for well-being and reducing distress

Participants found a variety of strategies and activities helpful in promoting overall well-being and reducing distress. The majority of these were leisure activities which would not necessitate health professional involvement but could be facilitated within community services or groups. Examples included exercise, art, spiritual activities, and volunteering to help others. These activities were largely characterized as providing distraction and keeping busy to keep one’s mind off the cancer. One participant found fulfillment in using their hobbies to give back to the people who support them. Experiences of fulfillment and rewarding social interactions were also shared by those who kept busy through volunteer work.

### Styles of coping with cancer

In talking about distress, this led many participants to talk about their attitudes toward their cancer diagnosis more broadly. Representation of different styles of coping emerged from this discussion. Subthemes included being positive, acceptance, and distancing; quotes are provided in Table 4.

**Table 4:** Styles of coping with cancer

Being positive
“I truly believe the more positive you are, the better off you are.” (Female, 53 head and neck cancer, DT = 7) “I think certainly being positive is a help because it’s like a slippery slide if you start thinking about, oh, I’ve got aches and pains, and it’s not going to get any better, and I’ve got… and you just slide down the slope. It gets worse and worse. If you’re positive, you might still go down the slide, but it’s not a quick run.” (Male, 70, prostate cancer, DT = 0) “Trying to stay positive, I guess, and not let things get to me too much. If I feel down, I guess I start to think about happy things.” (Female, 31, bowel cancer, DT = 0)
Acceptance
“‘I’ve sort of just accepted it as part of life and, of course, you have to learn to live with it.’ (Female, 75, bowel and liver cancer, DT = 4)
“I’m not happy about what is going on or how I feel in any event, but I’m quite resolved to the fact that this is what I’ve got.” (Male, 72, prostate cancer, DT = 6)
“I’ve my children and all that kind of thing, and I said, well they don’t need me anymore; everything is alright if I go.” (Female, 78, esophageal cancer, DT = 6)
“I’ve had a fair innings, and I’m quite happy to play the game as it goes.” (Male, 72, kidney cancer, DT = 5)
Distancing
“I don’t talk about that, no. I think if you would talk about that, mate, I think you’d end up with a very negative attitude.” (Male, 67, prostate cancer, DT = 8)
“See, I don’t think about it as what I’ve got is cancer. I try just to think that the… It sounds really, really silly. I know I’ve got tumors and I’ve got a lot of them, but I think of them as a tumor, not as a cancer. I tend to say the cancer word makes it more real, if that makes sense. So I just call them tumors. To me it doesn’t sound as bad.” (Female, 53, head and neck cancer, DT = 7)

#### Being positive

The first clear style of coping that appeared was being positive. Although centered on positive belief, this attitude was expressed as though it were an active stance, a committed notion of “fighting,” with the implication that not doing so (being positive) could lead to worse outcomes.

#### Acceptance

Another approach that participants described was acceptance.

While being a common thread, participants’ reasons for this acceptance were different: from knowing that family would be okay to acknowledging the life that they have already had.

#### Distancing

The final subtheme in styles of coping involved not thinking about the cancer. For some people, this also meant not talking about the cancer, or even naming it.

### Understanding of distress

Although we provided participants the NCCN definition of distress in our interviews, and they had recently been through the process of distress screening, there was some lack of understanding as to the meaning of distress. Participants separated their experiences of anxiety from the term “distress.” While distress is defined within research and clinically as encompassing common and normal feelings, participants saw this term as representing the more severe end of this continuum (see Table 5).

**Table 5:** Indicative quotations: understanding of distress

Understanding of distress
“I don’t think I really understand the term distress. I don’t think I’m distressed, and I don’t think I ever have been distressed, but maybe I have a different definition to distress than what is meant by you people. I don’t know… They aren’t worries. I feel a little bit anxious. But, yes, I don’t worry about it, and I certainly don’t get distressed about it. I would say if you’re distressed you’re virtually at a place where you just feel as though you can’t cope, and your whole world’s falling apart and so on. To me that is distress [unclear] almost permanently wounded.” (Male, 72, prostate cancer, DT = 6) “I remember the first time I did the distress, I thought this is a big word. Distress was quite a big word to be using because at that time, I’d just been diagnosed and I obviously hadn’t felt the effects of anything really yet, except probably the emotional side. But I wouldn’t say I was distressed at the time. Rather than distress I’d say I have anxiety sometimes…” (Female, 31, colorectal cancer, DT = 0) “That’s a bit of a problem because I’m not sure what part of the distress you want me to talk about, the distress about my chemotherapy problems, or my operation problems, or the coming operation problems, which is what I’m doing a pain chart for, now.” (Male, 75, bladder cancer, DT = 5)

## Discussion

This qualitative exploration revealed important information on the experience of brief distress screening among a group of people with cancer who had been screened as part of their usual appointment in an Australian hospital cancer clinic. This distress screening program is similar to other brief programs internationally [28, 29] and uses one of the most common screening tools (the distress thermometer) [21]. When considering the overall acceptability of the screening program, participants contextualized their distress within broader themes of coping, support from their personal networks, and questioned the definition of distress.

### Distress screening was generally acceptable to cancer outpatient participants

It is widely recognized that cancer diagnosis and treatment can significantly affect patients’ well-being, such that distress during cancer is now considered the sixth vital sign [11, 12]. An issue of clinical importance is that, despite the implementation of distress screening and the established evidence base for the effectiveness of psychological interventions to reduce distress, supportive care referral is generally low [30]. This has led to investigations to determine why this is the case. The findings of this study reaffirm that patients generally find distress screening to be acceptable although not always personally applicable [31]; furthermore, some participants did not perceive formalized supportive care services to be relevant or valuable.

The logistics of distress screening, particularly the timing and health professional involved, showed variable preferences. For some participants, if presented too soon or without sufficient explanation, distress screening was seen as not useful. In some cases, it was reported as overwhelming amidst the start of treatment with information and appointments. This echoes other study findings in which patients and clinicians felt screening was more effective middle to late in the cancer trajectory rather than early [32].

Participants endorsed distress screening as the role of numerous health professionals; no single consistent clinician type was identified, and our participants’ views demonstrate differing preferences for which clinicians should address distress. Clinical guidelines recommend that everyone responsible for the patient care should be at least aware of how the patient is progressing through the distress screening and management pathway [13]. However, an implementation barrier often cited is confusion as to roles and responsibilities in this process and the lack of time and confidence to ask about distress and provide follow-up [18, 20]. Developing specific roles and responsibilities for the members of the multidisciplinary team along with training modules would facilitate distress screening implementation models. This principle of allocating responsibility should also extend to referrals, whereby a member of the healthcare team ensures need-based referrals are made and patients are empowered to action the referral.

### Patients may not perceive supportive care referrals as personally relevant

Participants identified social supports such as family and friends to be paramount in providing support throughout their cancer diagnosis and treatment. For some participants, this was cited as being more important than formalized support. This may be one reason for low referral uptake, particularly among those who do not perceive themselves as distressed. Conversely, there was another group who felt that they did not want to burden their friends and family but saw value in talking about their experience. Availability and willingness to draw upon family and social support might be an important consideration when considering and presenting referral to patients. This notion has been explored previously in a study that highlighted that receptivity to referral is a separate issue from distress levels [33].

A further study suggests that referral uptake is driven, in part, by patients’ conceptualizing psychological support as preventative to worsening distress as opposed to reactive when distress is already severe [34]. Knowledge of the benefits of support was also associated with increased referral uptake [30]. Within our study, participants often did not feel supportive care services were required because they did not feel distressed “enough.” It is possible that this brief distress screening model did not provide patients with information on the support services nor the motivational coaching required to action supportive care referrals. In order to maximize the utility of screening, health professionals must confidently action screening results and empower the distressed patient to recognize the personal benefit of supportive care. This may require a paradigm shift as many health professionals are focused on the biomedical model of care, along with dedicated resources to provide timely access to embedded supportive care services [35]. The lack of training to confidently identify and manage distress is a common barrier reported by cancer professionals [36].

### The utility of distress screening across different patient coping styles

Interview participants noted oppositional coping styles, acceptance, and positivity versus distancing. This finding aligns with previous studies. For example, a qualitative study emphasized cognitive distancing as a coping strategy among cancer patients [37]. These different approaches have implications for both the method in which distress is introduced, measured, and then discussed by health professionals [37].

Individuals with distancing coping strategies may choose to opt-out of distress screening and provide a non-representative answer (i.e., provide a lower score), or health professionals may be reluctant to continue discussions about emotional well-being. A qualitative study exploring general practitioners’ perceptions of assessing distress in cancer patients identified “denial” as a barrier to implementing further psychosocial assessment [38]. A study of communication distancing with women with breast cancer suggests that coaching distant patients and their loved ones to have difficult conversations about emotional well-being may be an important psychosocial intervention to enhance coping capacity [39].

Acknowledging patients’ coping strategies is a complex component of providing person-centered care which requires patients’ preferences to be respected. For example, previous studies have demonstrated distant coping styles were positively associated with quality of life, whereas emotion-focused styles were negatively associated with quality of life [40]. Other studies have suggested distant thinking was related to worse long-term outcomes [41]. While this long-standing debate on the utility of distant coping continues [41], there is little guidance specifically on how to coach patients who are highly distressed and distant to utilize supportive care interventions. Furthermore, it would be valuable to explore the acceptability of psychosocial screening and uptake of subsequent referrals across coping styles in a larger sample using validated measures, such as the Mini-Mental Adjustment to Cancer or Brief-Cope Inventory. Also, it is important to acknowledge that while the coping styles have been presented as different approaches, it is likely that they are not mutually exclusive; patients may utilize different strategies depending on their evolving needs and experiences.

### Concept and definition of distress

Nomenclature surrounding mental health is challenging, and the term “distress” was selected by tool developers as it was perceived to be less stigmatizing than “psychological” or “emotional” and therefore more acceptable [3]. However, the term distress can have different meanings across different disciplines and areas of life [42]. As a one-item tool, the DT is perceived as efficient in quickly capturing individuals who are potentially experiencing distress. Nevertheless, it is paramount that those completing the tool have a clear understanding of the definition of distress. So that tools remain brief, it may be ideal to have a quick orientation to the concept of distress before the first administration or provide an abbreviated patient resource with a more extensive definition [43]. Future studies could explore the effect and patient experience of these suggestions.

## Limitations

This research may have been affected by selection bias. As part of the recruitment process, patients in the clinic were not invited to participate if they declined completing the DT. Other patients declined to participate citing not feeling they could contribute to a discussion about distress. It is possible that patients who declined the screening process or study participation may have differed from the study participants in their perceptions of being asked about distress, managing their diagnosis and ways of coping.

The study also included individuals who were newly diagnosed and those who were more than 2 years post diagnosis. Although this aligns with universal screening of all individuals in contact with the health service and the majority of our participants reported some level of distress, it is possible that individuals reflected differently on their experience of distress screening. We also did not ask participants if they had completed other emotional well-being screening questions or had previously completed the DT in other settings. As patient-reported outcome and experience measures become more integrated into health services, it is possible that patients particularly those who had been diagnosed more than 2 years ago had become more comfortable and familiar with completing these exercises.

Additionally, none of the participants in this study identified as being of Aboriginal or Torres Strait Islander descent. Although the DT is a validated tool, acceptability of screening tools can vary across and within cultures. Distress in Aboriginal and Torres Strait Islander populations remains under-researched [44], and further studies should consider acceptability and cultural safety in these populations.

It is important to note that this study discussed only one form of available distress screening methods which was not supported by a formalized referral pathway at the time of screening, though the cancer service did have access to a psycho-oncology team.

## Conclusions

This study found patients are generally accepting of in-clinic distress screening, and brief screening tools are important triggers or “red flags” for subsequent discussions. However, just as our study participants expanded discussions of distress screening to broader concepts of coping and support, so must health professionals. Our results suggest that in order for patients’ distress to be accurately captured and supportive care to be provided, clinicians and our systems must consider patients’: varying coping strategies, external support networks, understanding of terms, and motivations for accessing supportive care services. More research is needed to elucidate how we gather this information and how it impacts the distress screening process.

## Supplementary Information

Below is the link to the electronic supplementary material.

Supplementary file1 (PDF 514 KB)

## Data Availability

NA.

## Code availability

NA.
